# Regenerative Endodontic Procedure in Mandibular First Molar: A Clinical Case Report

**DOI:** 10.1002/ccr3.73610

**Published:** 2026-10-12

**Authors:** Sorour Saeedi, Ali Hamedi, Mani Mansouri, Iman Shiezadeh

**Affiliations:** ^1^ School of Dentistry Mashhad University of Medical Sciences Mashhad Iran; ^2^ Private Practice Mashhad Iran; ^3^ Student Research Committee, Faculty of Dentistry Mashhad University of Medical Sciences Mashhad Iran

**Keywords:** case reports, immature teeth, mandibular molar, nonvital tooth, periapical periodontitis, regenerative endodontics, tooth root

## Abstract

Regenerative endodontic procedures (REPs) have emerged as a biologically based treatment modality for managing immature permanent teeth with necrotic pulp, offering the potential for continued root development and periapical healing. This case report describes the regenerative endodontic treatment of a necrotic, immature mandibular first molar in a 9‐year‐old female patient. The treatment protocol involved canal disinfection using double antibiotic paste (DAP), induction of intracanal bleeding to form a natural scaffold, and coronal sealing with mineral trioxide aggregate (MTA). A 12‐month follow‐up demonstrated successful resolution of the periapical lesion, continued root maturation with increased root length and wall thickness, and clinical absence of symptoms. The extraoral sinus tract also healed completely within this period. This case highlights the clinical viability of REPs in immature multirooted teeth, emphasizing the importance of appropriate case selection, careful scaffold induction, and long‐term monitoring. Although short‐term outcomes were favorable, extended follow‐up is necessary to fully validate the durability of regenerative success.

## Key Clinical Message

Regenerative endodontics can be considered a reliable approach for the management of necrotic immature mandibular molars. As shown in this report, thorough canal disinfection combined with the induction of a blood clot can lead to the resolution of apical periodontitis and continued root development, even in complex multirooted teeth.

## 1 Introduction

Endodontic treatment of necrotic immature permanent teeth is still one of the most difficult procedures. Apexification of calcium hydroxide or mineral trioxide aggregate (MTA) has been a long‐established method for stimulating apical closure. Unfortunately, such treatments have a high risk of not inducing root growth or thickening of the dentin wall and leave a structurally flawed tooth with a high risk of fracture [1, 2]. In recent years, regenerative endodontic procedures (REPs) have emerged as a biologically based alternative with the potential to restore pulp‐like tissue and support continued root maturation. The conceptual foundation of REP relies on the triad of tissue engineering: stem cells, scaffolds, and signaling molecules. By inducing periapical bleeding, clinicians aim to introduce stem cells from the apical papilla (SCAP) into the canal space, where they interact with a scaffold (e.g., blood clot) and endogenous growth factors to facilitate healing and tissue regeneration [3].

Effective disinfection is essential for the success of the REP, requiring the elimination of intracanal bacteria while preserving the viability of apical stem cells. This balance is typically achieved through minimal mechanical instrumentation and passive irrigation with biocompatible solutions such as low‐concentration sodium hypochlorite and 17% EDTA [4]. The adjunctive use of intracanal medicaments like triple or double antibiotic pastes (TAP/DAP) further enhances disinfection while minimizing cytotoxic effects on regenerative tissues [5, 6].

Despite the increasing body of evidence supporting REP in anterior teeth, the application of this technique to immature molars is relatively underreported, and the available data regarding long‐term success rates in multirooted teeth remains limited [7]. Molars represent a significant procedural challenge compared to anterior teeth, as these teeth have intricate root canal anatomy and difficulty in creating and maintaining optimal intracanal bleeding for the stimulation of thrombus formation [8]. Nevertheless, recent studies have demonstrated that posterior teeth exhibit superior regenerative outcomes compared to anterior teeth, with a mean increase of 39% in root area and 11% in root length for posterior teeth, versus 26% and 6%, respectively, for anterior teeth, indicating statistically significant differences favoring posterior root development after the REP [9]. Based on these findings, the REPs represent a particularly suitable treatment option for pediatric patients presenting with immature posterior teeth, offering the potential for continued root development and long‐term tooth preservation. Patient age, especially in younger populations, has been suggested to significantly influence the regenerative potential of dental pulp tissues [10].

Considering the challenges in treating immature necrotic molars, regarding disinfection and scaffold formation, the REP provides a biologically based treatment alternative to retain natural dentition along with continued root development. This report aims to illustrate the application of REP in an immature mandibular molar and to discuss the critical clinical considerations that influence the success of such treatments, including proper case selection, scaffold induction strategies, and the need for diligent posttreatment monitoring. This report demonstrates that despite the anatomical complexities of mandibular molars, adequate disinfection combined with blood clot induction can result in substantial root maturation. The primary objective of this approach was to promote continued root development, specifically in terms of root length and dentinal wall thickness. Ultimately, achieving apical closure enhances the structural integrity and long‐term retention of the tooth, offering a distinct clinical advantage over conventional apexification.

## 2 Case History/Examination

A 9‐year‐old female patient presented with localized pain in the mandibular first molar. Clinical examination revealed tenderness on percussion and the presence of an extraoral sinus tract. The tooth had a history of restoration with amalgam. Her medical history was unremarkable, with prior pulpotomy and stainless‐steel crowns in primary molars. Radiographically, the mandibular first molar exhibited open apices, thin dentinal walls, and a periradicular radiolucency (Figure 1).

**FIGURE 1:**
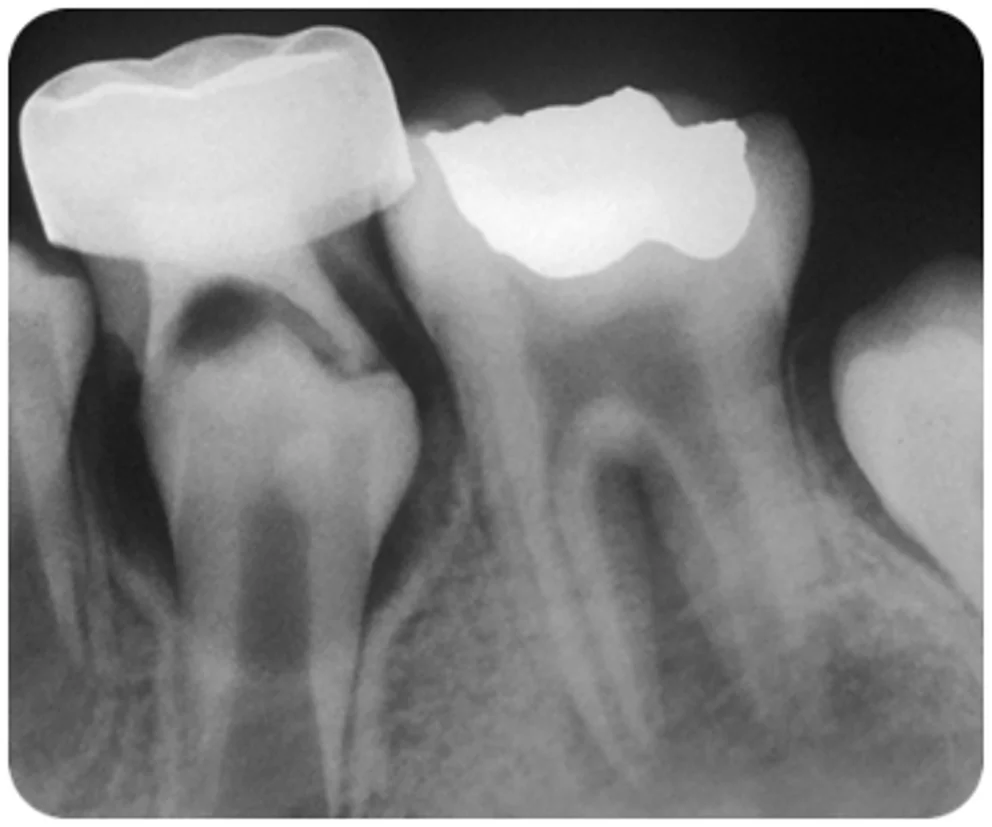
Preoperative radiograph showing an immature mandibular first molar with open apex and periapical radiolucency.

## 3 Differential Diagnosis, Investigations and Treatment

A comprehensive evaluation suggested that the patient was suffering from pulpal necrosis associated with a chronic apical abscess. Initial investigations included a periapical radiograph, which confirmed the presence of immature roots with open apices and a distinct radiolucent area at the periradicular region (Figure 1).

Working length was determined using #30 and #40 K‐files under radiographic control (Figure 2), ensuring accurate measurement while preserving apical tissues. Working length was determined radiographically, as electronic apex locators are generally unreliable in teeth with wide open apices.

**FIGURE 2:**
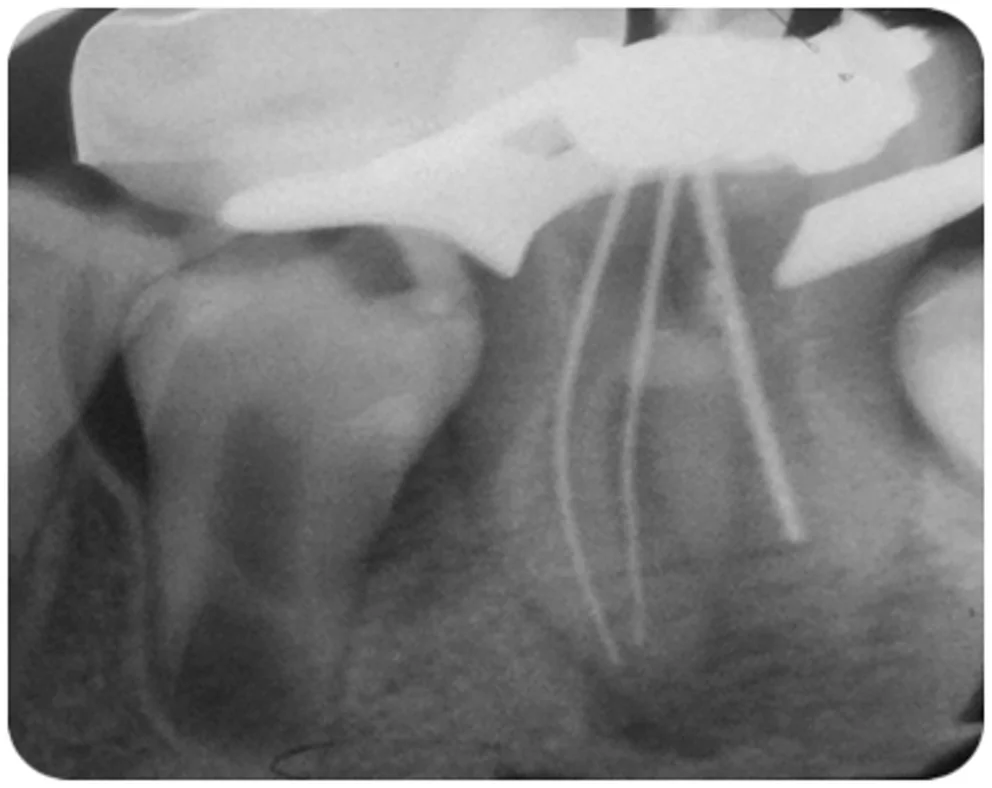
Radiograph showing working length determination using #30 and #40 K‐files.

The treatment protocol was executed in two visits, as summarized in the workflow chart (Figure 3). During the first visit, following an inferior alveolar nerve block with 1.8 mL of 2% lidocaine containing epinephrine (1:100,000), rubber dam isolation and access cavity preparation were performed. Mechanical instrumentation was kept to a minimum, using ISO #30 and #40 K‐files to avoid disruption of the apical stem cell niche. The canals were irrigated with 1.5% sodium hypochlorite followed by saline, and a double antibiotic paste composed of ciprofloxacin and metronidazole was placed as an intracanal medicament before sealing the tooth temporarily.

**FIGURE 3:**
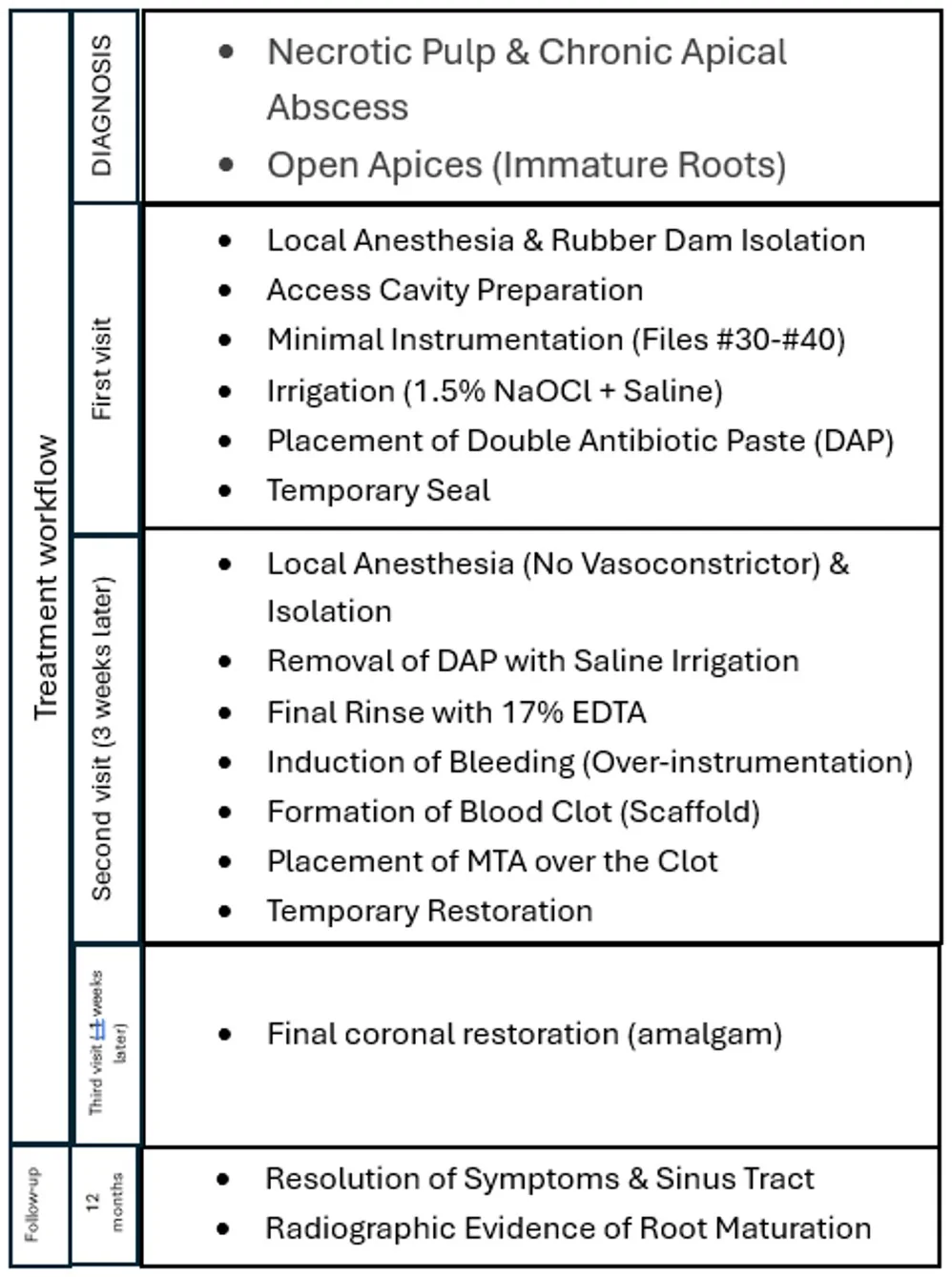
Treatment flowchart.

At the second visit, 3 weeks later, anesthesia was achieved using 3% mepivacaine without a vasoconstrictor. The DAP was removed with minimal instrumentation and irrigation with saline. This was followed by a 17% EDTA rinse (Cerkamed, Poland) to condition the dentin and facilitate the release of growth factors. A final flush with saline was then performed. Intentional bleeding was induced by gently over‐instrumenting approximately 1–2 mm beyond the apex with a sterile #25 K‐file to promote the formation of a blood clot within the canal, which would serve as a natural scaffold rich in growth factors and stem cells. Subsequently, Mineral Trioxide Aggregate (MTA) (Angelus, Brazil) was placed approximately 3 mm below the cementoenamel junction to serve as a biocompatible coronal seal. Proper placement of the MTA was confirmed radiographically (Figure 4). A moist cotton pellet was placed over the MTA, and the cavity was sealed with a temporary restorative material. The patient was then referred for final coronal restoration. Given the extensive loss of coronal tooth structure, a permanent amalgam restoration was placed to ensure a reliable coronal seal.

**FIGURE 4:**
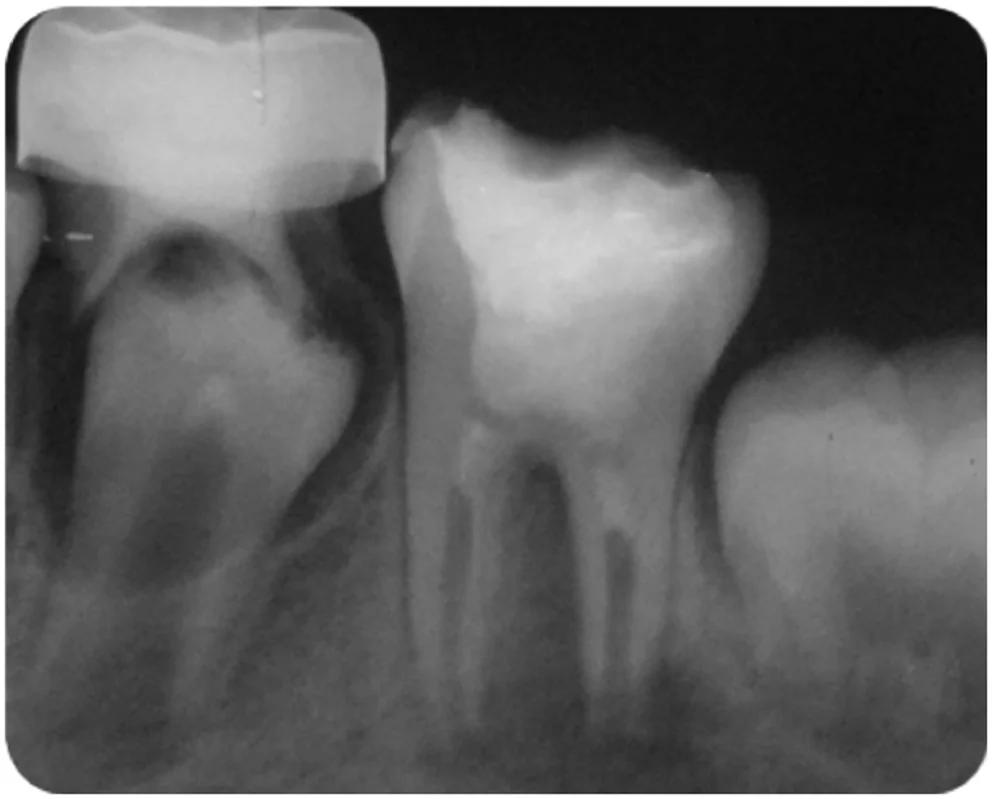
Postoperative radiograph demonstrating placement of Mineral Trioxide Aggregate (MTA) as a coronal barrier.

## 4 Results (Outcome and Follow‐Up)

At the 12‐month follow‐up, the patient was asymptomatic, and the extraoral sinus tract had completely resolved. Radiographs demonstrated healing of the periradicular lesion, continued root maturation with increased dentinal wall thickness, and evidence of apical closure (Figure 5). Clinical examination confirmed soft tissue healing (Figure 6a,b). A summary of clinical findings and pulp sensitivity tests is provided in Table 1.

**FIGURE 5:**
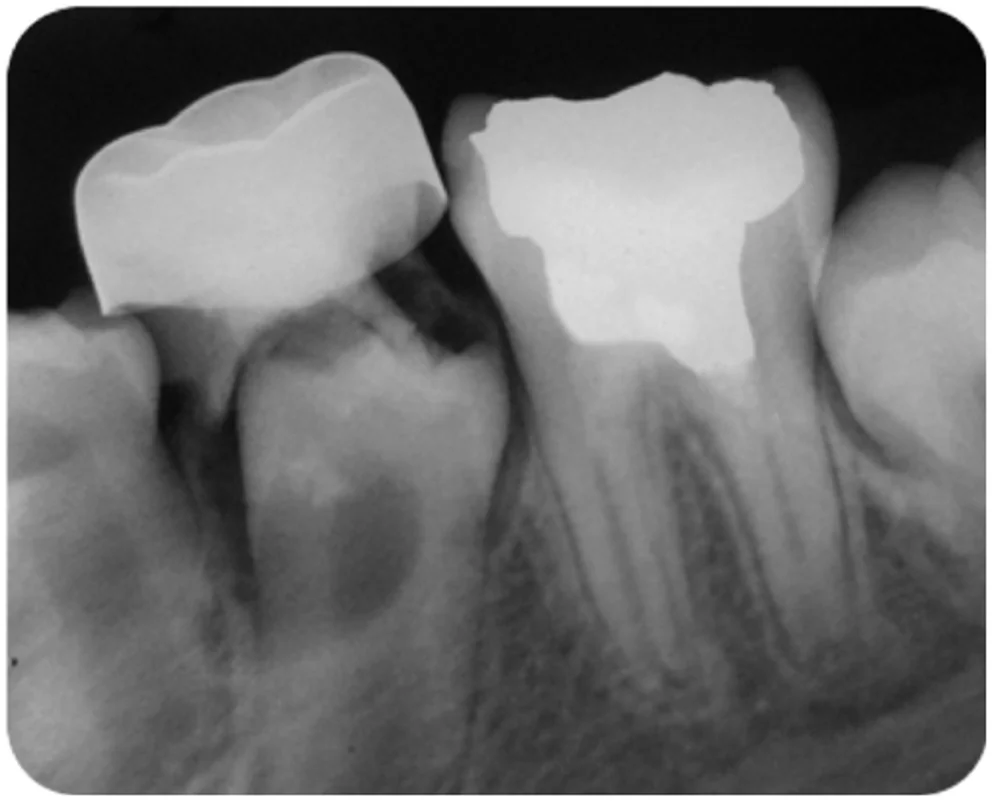
Twelve‐month postoperative periapical radiograph showing resolution of the periapical lesion, continued root development, and thickened dentinal walls.

**FIGURE 6:**
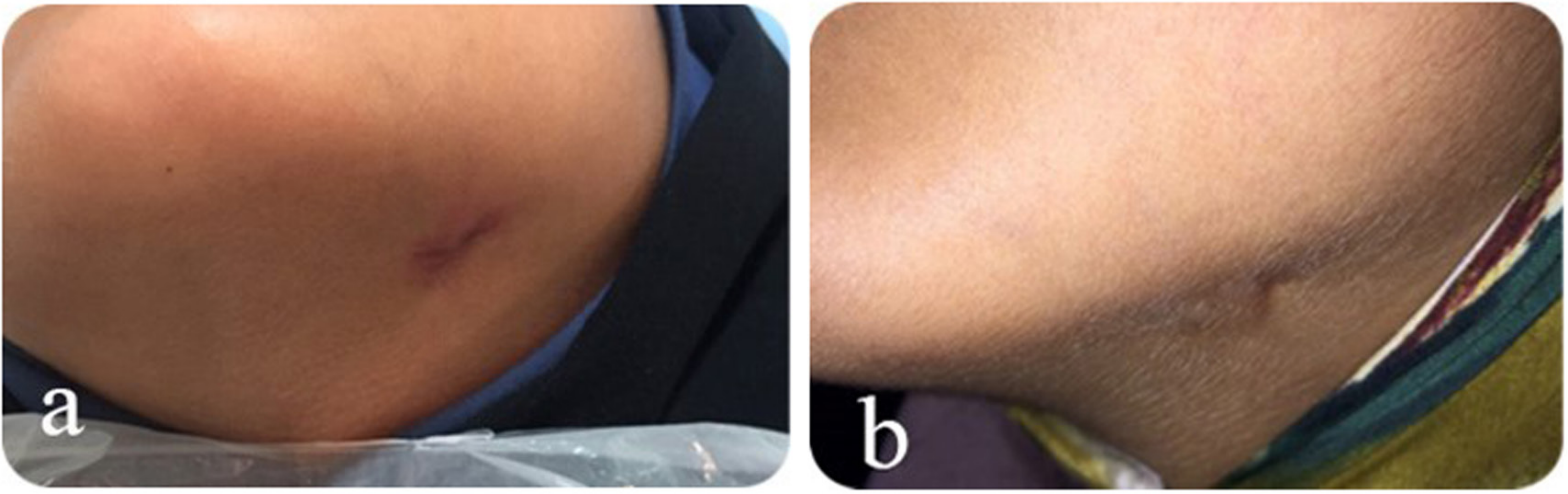
(a) Extraoral clinical photograph at the initial visit showing a draining sinus tract on the right side of the chin, indicative of chronic apical infection. (b) Extraoral image at 12‐month follow‐up demonstrating complete soft tissue healing and closure of the sinus tract.

**TABLE 1:** Chronological summary of clinical findings and pulp sensitivity tests for the mandibular right first molar during regenerative endodontic treatment.

Session	Mobility (1–3)	Periodontal status (0–1)	Palpation (0–3)	Percussion (0–3)	Cold test (0–4)	Heat test (0–4)	E.P.T. (0–10)
Baseline	1	0	2	1	0	0	0
Second visit	0	0	0	0	1	1	5
Mid follow‐up	0	0	0	0	1	0	6
Final visit	0	0	0	0	0	1	4

Abbreviation: E.P.T., Electric Pulp Test.

## 5 Discussion

Regenerative endodontic procedures (REPs) have emerged as a biologically driven alternative to conventional apexification techniques for treating immature permanent teeth with necrotic pulp. This case demonstrates the effectiveness of using REP in a necrotic immature mandibular molar that led to root maturation and periradicular healing after inducing intracanal bleeding. Although REP has shown promising outcomes in many studies, particularly for anterior teeth, its use in posterior multirooted teeth remains less common and often presents greater technical challenges [11].

It is crucial to know what contributes to these outcomes, particularly since the anatomical complexity of the posterior teeth is already challenging enough. Among the factors determining the success of REPs, the patient's age was consistently recognized as one of the most important. Younger patients, particularly those under the age of 14, exhibit greater regenerative capacity due to higher concentrations of viable stem cells from the apical papilla (SCAP) and enhanced angiogenic potential. Smoczer et al. [12] demonstrated that stem cells from the apical papilla retain high viability and migratory potential even when exposed to calcium silicate‐based materials. These stem cells also show faster doubling times and strong responsiveness to growth factors such as VEGF, PDGF, and TGF‐β1, making them especially suited for regenerative applications in younger patients [13, 14, 15]. This biological advantage is further supported by findings from Jiang and Liu [15], whose large‐scale retrospective study revealed that younger age at the time of treatment was significantly associated with achieving both primary and secondary regenerative outcomes (*p* < 0.05).

Another critical factor influencing regenerative outcomes is the presence of an open apex. A wide apical foramen facilitates the influx of stem cells from the periapical region and promotes sufficient intracanal bleeding to serve as a biological scaffold. According to Theekakul et al. [16], a larger apical diameter was significantly associated with higher rates of apical closure, with a reported mean reduction of 56.8% in foramen size following REP. Notably, 26.7% of immature teeth treated showed complete closure of the apex on follow‐up, and the best outcomes occurred when patients were younger than 12 years old.

However, anatomical structure alone does not always favor regeneration. Complementing this, a micro‐CT study by Hristov et al. [17] revealed that immature molars often display irregular or ribbon‐shaped canal configurations in the apical region. Depending on the individual case, these complex morphologies can either facilitate regenerative healing or introduce technical challenges. Such variations underscore the importance of meticulous case selection and irrigation control, as both are vital to minimizing the risk of irrigant extrusion and ensuring a stable environment for tissue growth. While anatomical variation is a natural feature of immature molars, their multirooted structure and curved canal systems pose considerable challenges for regenerative procedures. These features can limit effective disinfection and make it more difficult to uniformly induce bleeding or maintain scaffold stability across all canals. Shojaeian et al. [18] emphasized that incomplete elimination of intracanal infection remains a leading cause of failure in immature molars undergoing revascularization, largely due to their complex root morphology.

In spite of the above‐mentioned issues, clinical outcomes for posterior teeth have been increasingly predictable. Alfahadi et al. [19] reported satisfactory clinical and radiographic outcomes in posterior teeth treated with conventional REP protocols, substantiating the fact that molars' regeneration is practicable and effective with proper case selection and adherence to guidelines.

This was a case with a number of positive factors present that most likely ensured success for the regenerative treatment. The patient was 9 years old and had an immature mandibular first molar with an open apex and thin dentinal walls, perfect for migration of stem cells and root development. Having neither systemic health problems nor a history of trauma nor past root canal treatment further reduced confounding factors.

From a procedural perspective, certain clinical decisions may have also served to enhance the regenerative outcome. Intracanal bleeding was intentionally induced using a sterile #25 K‐file, which enabled a natural clot to be developed as a scaffold for the migration and regeneration of cells. Unlike synthetic or engineered alternatives, this approach provides an easy, biocompatible matrix that is consistent with present tissue engineering principles. Additionally, the use of double antibiotic paste (DAP) in place of triple antibiotic paste (TAP) obviated the risk of coronal discoloration, but still afforded effective canal space disinfection.

When placed against the context of conventional techniques, the benefits of REP are more pronounced. While traditional techniques such as apexification with Calcium Hydroxide (Ca(OH)_2_) or Mineral Trioxide Aggregate (MTA) have long been used, they are limited in promoting continued root development and dentinal wall thickening. Apexification with calcium hydroxide requires multiple visits, and while it causes closure of the apex, it makes the dentinal walls fragile and causes roots to be shortened [20]. Likewise, MTA apexification, though causing formation of the apical barrier quickly, also does not provide support for further development of the root or thickening of dentinal walls, thus compromising the tooth structurally [20]. However, regenerative treatments like REP support closure of the apex plus healing of the periapical region but also actual development of the root. Almalki et al. [21] reported significant increases in both root length and dentin thickness over a 5‐year period following REP, highlighting its biological superiority over apexification. The intracanal medicament also plays a crucial role in the overall success of regeneration. While TAP has been popular, its use of minocycline poses the risk of tooth discoloration and cytotoxicity. DAP, by removing minocycline and using ciprofloxacin plus metronidazole, ensures antimicrobial activity without causing excess side effects. Pekpinarli et al. [5], through a comparative study, reported equal efficacy of DAP for induction of root maturation and healing of the apex, thus proving to be a clinically safer option. The research indicates that the combination of DAP and intentional bleeding yields an advanced option for regenerative endodontic treatment for immature teeth, which yields better structural and functional results when compared to conventional apexification techniques.

In this case, scaffold formation was done through the induction of intracanal bleeding, which resulted in a natural clot that was a simple and biologically similar matrix for regeneration. Though widely practiced clinically, this technique has been joined by other scaffolds, which include platelet‐rich plasma (PRP) and platelet‐rich fibrin (PRF) for the same purpose. These blood‐derived concentrates are full of growth factors that enhance the proliferation and repair of cells [22]. According to Saldanha et al. [23], it was noted that the use of PRP and PRF was linked to increased root lengthening when compared to using a blood clot, but the induction of a blood clot showed better properties for closure of the apical foramen and equal effects on thickness of the root.

The simplicity, accessibility, and cost‐effectiveness of blood clot induction make it a practical option for many clinical settings. Nonetheless, PRP and PRF could be particularly useful for situations where a primary treatment concern is to obtain the highest amount of root elongation. Utilization, however, will be limited by the requirement for specialized apparatus, increased procedural complexity, and higher cost.

Although the 12‐month follow‐up in the present case demonstrated favorable clinical and radiographic outcomes, it must be acknowledged that this represents a short‐term observation. Long‐term follow‐up is critical, as studies such as Alharbi et al. [24] have shown that regenerative endodontic treatments can initially succeed but still develop complications like recurrent apical periodontitis several years after treatment. Similarly, long‐term data by Jothish et al. [13] emphasized that while high initial success could be detected, complications like reinfection and structural failure may occur over time, demonstrating the crucial nature of follow‐up visits. These findings indicate the necessity for continued follow‐up for complete appraisal of the durability and predictability of regenerative outcomes. This report has a few limitations. As a case report, the findings are limited to a single patient and cannot be generalized without larger clinical trials. Histologic evaluation of the newly formed tissue was not possible in a clinical setting.

## 6 Conclusions

At the 12‐month follow‐up, the patient was asymptomatic, and the extraoral sinus tract had completely resolved. Radiographic examination showed progressive healing of the periapical lesion, continued root maturation, and evidence of apical closure.

This case supports the potential of regenerative endodontic procedures as a biologically based treatment for immature necrotic mandibular molars. Successful outcomes in such cases depend on proper case selection, effective canal disinfection, bleeding induction, and a durable coronal seal. Although these short‐term results are favorable, long‐term follow‐up is necessary to confirm the stability of the treatment.

## Author Contributions

**Sorour Saeedi:** conceptualization, data curation, formal analysis, methodology, project administration, supervision, validation, visualization, writing – original draft, writing – review and editing. **Ali Hamedi:** conceptualization, project administration, resources, writing – original draft, writing – review and editing. **Mani Mansouri:** conceptualization, data curation, methodology, project administration, resources, software, writing – original draft, writing – review and editing. **Iman Shiezadeh:** conceptualization, data curation, methodology, project administration, resources, software, supervision, writing – original draft, writing – review and editing.

## Funding

The authors have nothing to report.

## Consent

Written informed consent was obtained from the patient for the publication of this case report and any accompanying images.

## Conflicts of Interest

The authors declare no conflicts of interest.

## Data Availability

The data that support the findings of this study are available from the corresponding author upon reasonable request. The data are not publicly available due to privacy or ethical restrictions.
